# Numerical Analysis of the Relationship between Friction Coefficient and Repose Angle of Blast Furnace Raw Materials by Discrete Element Method

**DOI:** 10.3390/ma15030903

**Published:** 2022-01-25

**Authors:** Shiyu Wei, Han Wei, Henrik Saxen, Yaowei Yu

**Affiliations:** 1 State Key Laboratory of Advanced Special Steel, Shanghai Key Laboratory of Advanced Ferrometallurgy, School of Materials Science and Engineering, Shanghai University, Shanghai 200240, China; shiyuwei@shu.edu.cn (S.W.); weihan@shu.edu.cn (H.W.); 2 Process and Systems Engineering Laboratory, Faculty of Science and Engineering, Åbo Akademi University, Henriksgatan 8, FI-20500 Åbo, Finland; Henrik.Saxen@abo.fi

**Keywords:** discrete element method (DEM), repose angle, particle, friction coefficient

## Abstract

In recent years, the discrete element method (DEM) has been widely used to study the factors affecting the repose angle and calibrate particle parameters for simulations. In this paper, DEM is used to study the effects of the coefficient of rolling and static friction of pellet, sinter and coke particles on the repose angle. By comparison of the results of simulations and physical experiments, the coefficients of rolling and static friction suitable for simulation work are determined. The results demonstrate that repose angle increases with the coefficient of rolling and static friction, but the rate of increase gradually decays, when the coefficient of rolling friction exceeds 0.4 or the coefficient of static friction exceeds 0.35. The coefficient of static friction has a greater impact on the repose angle than the coefficient of rolling friction. The rougher of the base surface, the larger the repose angle of the formed particle piled. It can be concluded that appropriate coefficient of rolling and static friction for simulations can be obtained by the outlined procedure.

## 1. Introduction

Granular material, such as soil, sugar, grain and gravel, widely exists in nature, daily life and engineering applications. Because of such common occurrence, people are curious about the accumulation form and law of particles, such as the exploration of avalanche [1], use of hourglass timing and study of “granary effect” [2]. In the flow of granular material, after a large number of irregularly moving single particles gather together, the particle flow establishes a regular motion. Further analyses reveal that granular material exhibit properties similar to but not completely consistent with traditional solids and fluids. For example, the particles are solid, but when the particle pile collapses, a large number of particles could flow like a fluid. Traditional solid and fluid theory cannot explain this phenomenon [3].

The repose angle is an important feature of granular material at accumulation and usually refers to the maximum inclination angle formed by the granular material pile relative to the horizontal plane, in which the particles can accumulate without collapse [4]. The repose angle plays a key role in the storage, transportation, and processing of granular material. For the ironmaking blast furnace, the repose angle of burden layer is important to prevent the charge from collapsing and sliding on the burden surface [5]. Hence, it is very important to study the influence of the physical parameters of blast furnace raw materials on the repose angle.

A large number of studies have shown that the factors affecting the repose angle include water content [6], initial aspect ratio [7], particle shape [8] and size [9], coefficient of restitution [10], particle number [11], geometry [12] and friction coefficients [8,13,14]. It was also found that the properties of granular materials [15] largely affect the repose angle. For the friction coefficient, there are some arguments about the effect. The widely recognized dependence is that the repose angle increases with the friction coefficient and eventually approaches an asymptotic value [14]. However, there are indications [8] that the repose angle is slightly reduced when the friction coefficient becomes large, but without no reasonable explanation. Therefore, the influence of the friction coefficients on the repose angle needs more detailed research.

To study the repose angle, it is difficult to rely on physical experiments [16,17] alone due to the diversity of particle properties and the complexity of pile forming mechanism, and the variables are difficult to control accurately. Because of the ever-increasing computational power of computers, the numerical simulations have become very popular and is widely used to study the behavior of granular flow [18,19,20,21]. Many important methods and findings originate from the study of repose angles, including the particle sphere physical model [22], the study of rolling friction in sand pile formation [23] and the evaluation of rolling resistance model [24].

Cundall and Strack [25] were the first to propose the Discrete Element Model (DEM). The simulation method is commonly used in object dynamics due to its transparent and effective characteristics. DEM can be used to understand the microscopic movement of particles and the connection with the macroscopic particle flow. After determining the calculation model, selecting the physical geometry and initial conditions, the physical parameters have to be selected appropriately as they are very important for the accuracy of the simulation results by DEM. At present, in addition to laboratory measurements [26,27], the use of DEM to estimate physical parameters has become an established method [28,29,30]. However, the DEM parameters of blast furnace raw materials (pellet, sinter and coke) have not been well characterized. In particular, the friction coefficients for particle–particle and particle–wall contact have not been accurately determined despite their importance for the accuracy of the simulations.

Several investigators [8,14] have proposed that the particles shape affects the repose angle, and have demonstrated this by showing the similarity between repose angles in simulation with spheres and with real particles in experiments. However, it is very difficult to accurately model irregular particles. To solve this problem, Wensrich and Katterfeld [31] considered using friction coefficient to replace the influence of shape. The results showed that in simple cases, using the coefficient of rolling friction (CORF) as a “tunable parameter” to spherical particles can implicitly consider the effect of shape. Later, Pasha et al. [32] further demonstrated the effect of manipulating CORF to simulate the movement of non-spherical particles. Therefore, spherical particles can be replaced by non-spherical particles using modified CORF.

To study the relationship between friction coefficient and repose angle, and to obtain values of the friction coefficient for simulating the blast furnace raw material behavior, this work uses pellet, sinter and coke as materials. The influence of the CORF and coefficients of static friction (COSF) for particle–particle and particle–steel plate on the repose angle is studied through DEM simulation combined with physical experiments. By this procedure, appropriate values of CORF and COSF can be estimated for future simulations of the burden behavior in the blast furnace process.

## 2. Methodology

### 2.1. Experimental Method

#### 2.1.1. EDEM

EDEM is software implementing the DEM algorithm, which can calculate the motion of granular material. The DEM describes the motion of single particles in the particle system to calculate the overall state of the particle flow. This method considers two types of motion: translation and rotation, expressed by Newton’s second law of motion. The elastic contact model used in this work is the Hertz-Mindlin (no slip) model with RVD Rolling Friction compatibility model [33,34,35], adopting a Coulomb law of friction. This model is a commonly used in DEM simulations due to its accuracy and efficiency in force calculation.

**Figure 1:**
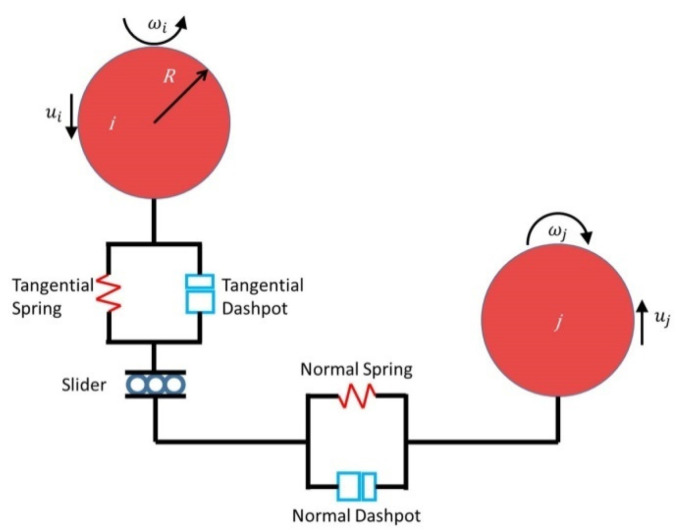
Contact force diagram of discrete element particles *i* and *j*.

As shown in Figure 1, the Hertz-Mindlin model uses the governing equations to describe the interaction between a particle (*i*) and another particle (*j*) in terms of translation (Equation (1)) and rotation (Equation (2)). The former includes normal contact force (*F_Cn,ij_*), tangential contact force (*F_Ct,ij_*), corresponding viscous damping force (*F_dn,ij_*, *F_dt,ij_*) and gravity (*m_i_g*). The latter represents the friction torque, including the torque generated by the tangential force ($M_{r}^{k}$) and torque generated by rolling friction ($M_{r}^{d}$). Equations (1)–(8) are the main expressions, which hold the coefficients and physical parameters involved in the model. In these, *k_n_* is the normal and *k_t_* is the tangential contact constant, *γ_n_* is the normal damping and *γ_t_* is the tangential contact damping constant. *Y*, *υ*, and *e*, in turn, represent the Young’s modulus of particles, Poisson’s ratio, and the coefficient of restitution between particles. As for friction coefficients, $\mu_{s}$ and $\mu_{r}$ represent COSF and CORF, while *F_t_* is the tangential and *F_n_* is the normal force. *R_i_*, *R_j_*, and *m_i_*, *m_j_* represent the radius and mass of particles *i* and *j*, *δ_n_* is the amount of deformation between particles, *I_i_* is the moment of inertia, $u_{i}$ is the transmission speed while $\omega_{i}$ is the rotation speed.

(1) $$m_{i}\frac{{du}_{i}}{dt}=\overset{K}{\underset{j=1}{\sum }}(F_{Cn,ij}{+F}_{dn,ij}{+F}_{Ct,ij}{+F}_{dt,ij}{)+m}_{i}g$$

(2) $$I_{i}\frac{{d\omega }_{i}}{dt}=\sum_{j=1}^{K}{(M}_{r}^{k}{+M}_{r}^{d})$$

(3) $$k_{n}=\frac{4}{3}\cdot \frac{Y_{i}Y_{j}}{Y_{j}\left(1-\vartheta_{i}^{2}\right){+Y}_{i}\left(1-\vartheta_{j}^{2}\right)}\sqrt{\frac{R_{i}R_{j}}{R_{i}{+R}_{j}}\delta_{n}}$$

(4) $$k_{t}=\frac{{4Y}_{i}Y_{j}}{Y_{j}\left(2-\vartheta_{i}\right)\left(1+\vartheta_{i}\right){+Y}_{i}(2-\vartheta_{j})(1+\vartheta_{j})}$$

(5) $$\gamma_{n}=-2\sqrt{\frac{5}{6}}\frac{ln(e)}{\sqrt{{ln}^{2}\left(e\right){+\pi }^{2}}}\sqrt{2\frac{Y_{i}Y_{j}\sqrt{\frac{R_{i}R_{j}}{R_{i}{+R}_{j}}\delta_{n}}\cdot \frac{m_{i}m_{j}}{m_{i}{+m}_{j}}}{Y_{j}\left(1-\vartheta_{i}^{2}\right){+Y}_{i}\left(1-\vartheta_{j}^{2}\right)}}$$

(6) $$\gamma_{t}=-2\sqrt{\frac{5}{6}}\frac{ln(e)}{\sqrt{{ln}^{2}\left(e\right){+\pi }^{2}}}\sqrt{\frac{{4Y}_{i}Y_{j}\sqrt{\frac{R_{i}R_{j}}{R_{i}{+R}_{j}}\delta_{n}}\cdot \frac{m_{i}m_{j}}{m_{i}{+m}_{j}}}{Y_{j}\left(2-\vartheta_{i}\right)\left(1+\vartheta_{i}\right){+Y}_{i}(2-\vartheta_{j})(1+\vartheta_{j})}}$$

(7) $${\;F}_{t}\leq \mu_{s}F_{n}$$

(8) $${|M}_{r,t+\Delta t}^{k}|\leq M_{r}^{m}=\mu_{r}\frac{R_{i}R_{j}}{R_{i}{+R}_{j}}F_{n}$$

#### 2.1.2. Measurement Method

In the measurement of the repose angle, the most commonly applied method is to use a protractor or similar tool to measure the angle manually [36]. This method is simple to operate, but human subjectivity may affect the accuracy of the results. Therefore, current measurement methods apply algorithms developed based on image analysis and coordinate measurement technology [36,37]. Many investigators [38,39] have determined the mechanical properties of materials by processing images. The general steps can be simply summarized to include binary processing, contour extraction, coordinate mapping and Gaussian fitting. The purpose of binary processing is to reduce the amount of data in the image and highlight the target contour. Contour extraction extracts contour coordinates on the basis of binarization. Coordinate mapping coordinates the data to draw contour lines by software. Finally, to make the profile of the particle pile smoother, the Gaussian function can be used to fit the contour to a bell curve.

For physical experiments in this work, we use photographs taken of the pile shape from four different directions with a fixed camera at the same vertical level as the center of the pile, and then determine the repose angle by software processing. For simulations, we use the angle measurement tool of EDEM post-processing to get the repose angle. Finally, a comparison of the bell-shaped curves of the simulations and physical experiments obtained by image processing is undertaken, and appropriate simulation parameters are determined to reproduce the experimental results.

### 2.2. Physical Experiment

**Figure 2:**
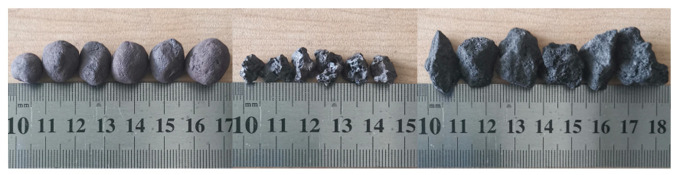
Particle size in physical experiments.

The pellet, sinter, and coke particles used in the experimental study are derived from blast furnace charge, and their dimensions are shown in Figure 2. Table 1 shows the parameters of the three kinds of particles used in the experiments. The equivalent spherical diameter (ESD) was used to represent the particle size due to the irregular shape. We used sieves with different meshes to determine the ESD range of the particles. The experimental method to form a pile is the lifting cylinder method [40], which is used to determine the repose angle. The method places a funnel filled with a proper number of particles on a base with known roughness, then lifts the funnel at a constant rate to form a pile, and finally measures the inclination angle of the particle pile as the repose angle.

**Figure 3:**
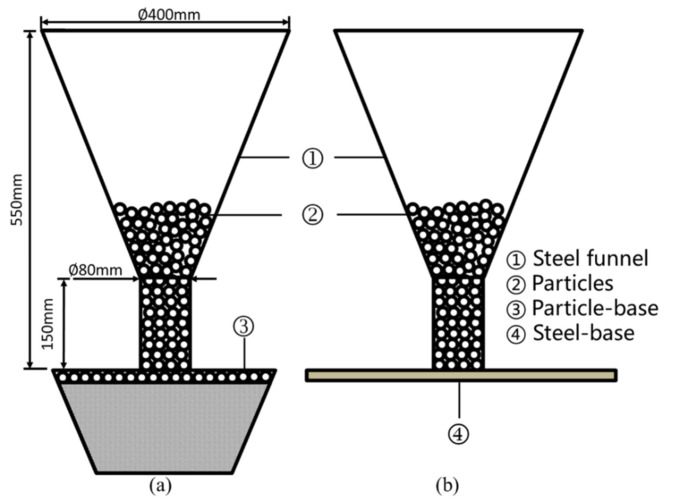
The geometric model of the experiments: (**a**) Particles-base. (**b**) Steel-base.

A schematic diagram of experimental device is shown in Figure 3. The experimental device of Figure 3a was used to study the repose angle of particle piles formed on a particle base, where a heap is formed on a layer of particles. The experimental groups studied include pellet–pellet (P-P), sinter–sinter (S-S), coke–coke (C-C) and pellet–coke (P-C). As an example, the P-C group uses P as the particle in the funnel, while C is the particle forming the base. The repose angles of pellet, sinter and coke piles on a steel base (P-Steel, S-Steel and C-Steel) were also studied, using the device shown in Figure 3b. Firstly, the steel funnel was placed in the center of the base, and the funnel was filled with particles. Secondly, the funnel was manually lifted to a position just higher than the final particle pile apex at a speed of 0.3–0.5 m/s. Photos at the front view of the stationary particle pile were taken, and the experiment was repeated ten times. Finally, the ruler tool of Photoshop was applied to measure the repose angle of the piles.

### 2.3. Simulation

Four experimental materials are involved in the experiments: pellet, sinter, coke and steel plate. Their basic parameters are shown in Table 2 [27]. This work only changes CORF and COSF of one contact surface of the experimental group. The friction coefficients of other contact surfaces are fixed and the parameters are shown in Table 3 [27].

The simulation process is the same as in the physical experiment. In the process of the simulations, CORF and COSF between particle–particle and particle–steel plate are changed. CORF is varied from 0.05 to 0.55 at intervals of 0.1, while CORF is varied from 0.1 to 0.6 at intervals of 0.1.

**Figure 4:**
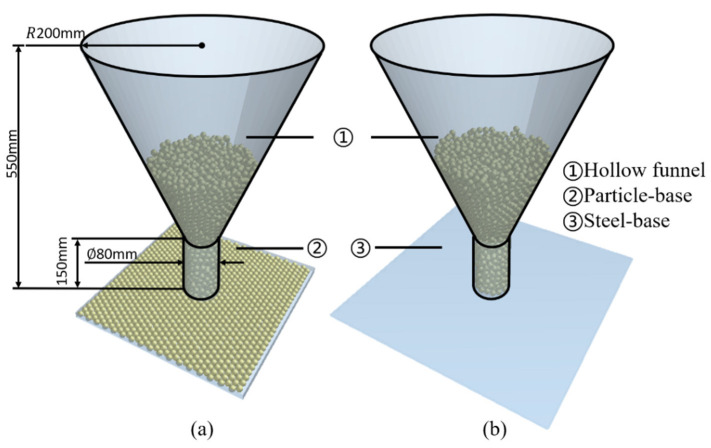
The geometric model of the experiments: (**a**) Particles-base. (**b**) Steel-base.

The experimental steps are as follows: Particles are randomly generated in the funnel, as shown in Figure 4. After the particles in the funnel have stabilized, the funnel was raised in the positive direction of the *z*-axis at a speed of 0.4 m/s to the height above the apex of the particle pile that forms. The time step in the simulations was set to 1.5 × 10^−5^ s. After the simulations, the repose angle in the four directions of ±x and ±y axis of the particle pile was measure and recorded, and average values were taken.

## 3. Experimental Results and Discussion

### 3.1. Simulation Results

#### 3.1.1. Selection Appropriate Lifting Funnel Speed

**Figure 5:**
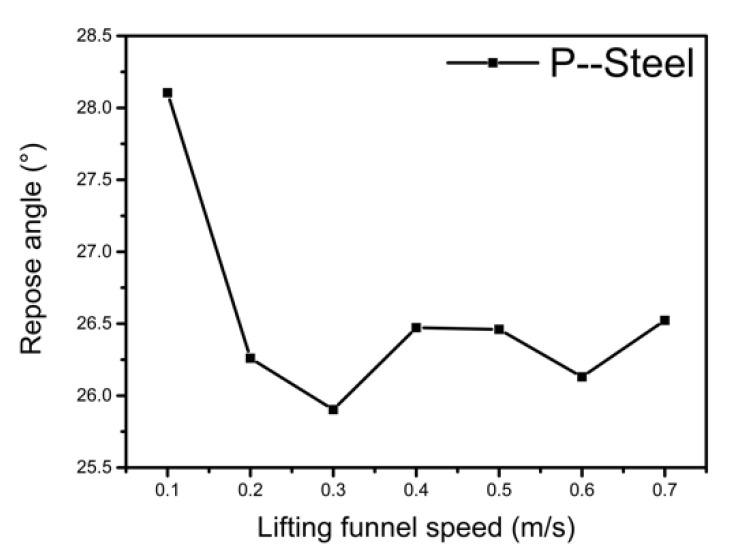
Relationship between the repose angle and the lifting funnel speed.

In physical experiments, due to insufficient accuracy of speed of the manual lifting of the funnel, the influence of the lifting speed on the repose angle must be considered before choosing an appropriate speed. The lifting speed of the funnel for P-Steel (CORF = 0.6, COSF = 0.55) was varied from 0.1 m/s to 0.7 m/s at intervals of 0.1 m/s, with results shown in Figure 5.

Analyzing Figure 5, it can be seen that the fluctuation of the repose angle is less than 1°, and within the error range, when the lifting funnel speed is 0.2–0.7 m/s. Therefore, the lifting funnel speed of the physical experiments is controlled to be 0.3–0.5 m/s, while the simulation setting is 0.4 m/s.

#### 3.1.2. Repose Angle of Different Blast Furnace Raw Materials

**Figure 6:**
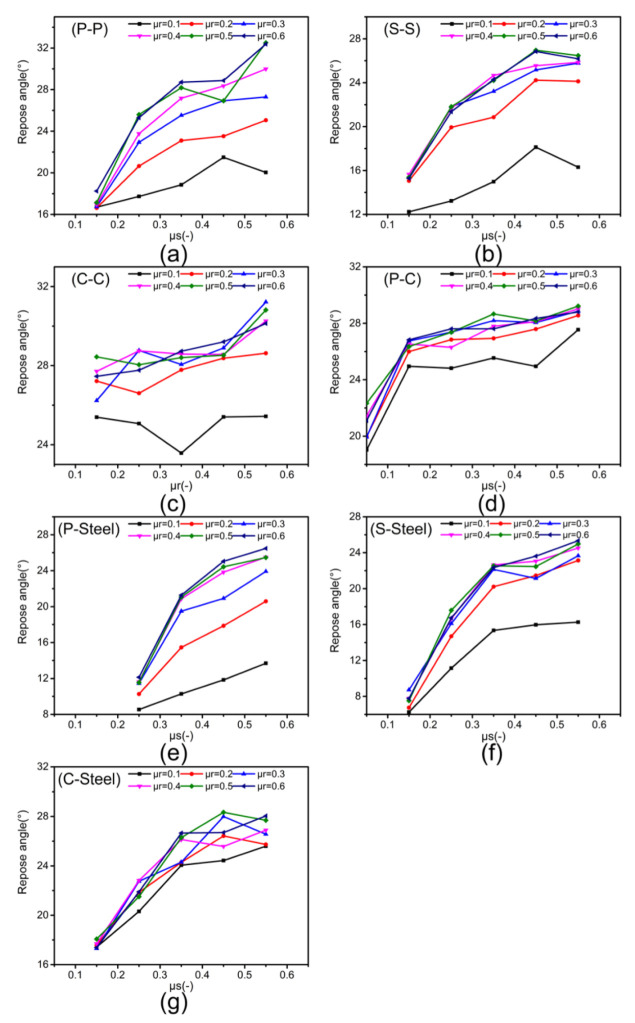
Relationship between repose angle, CORF, and COSF: (**a**) P-P, (**b**) S-S, (**c**) C-C, (**d**) P-C, (**e**) P-Steel, (**f**) S-Steel, (**g**) C-Steel.

**Figure 7:**
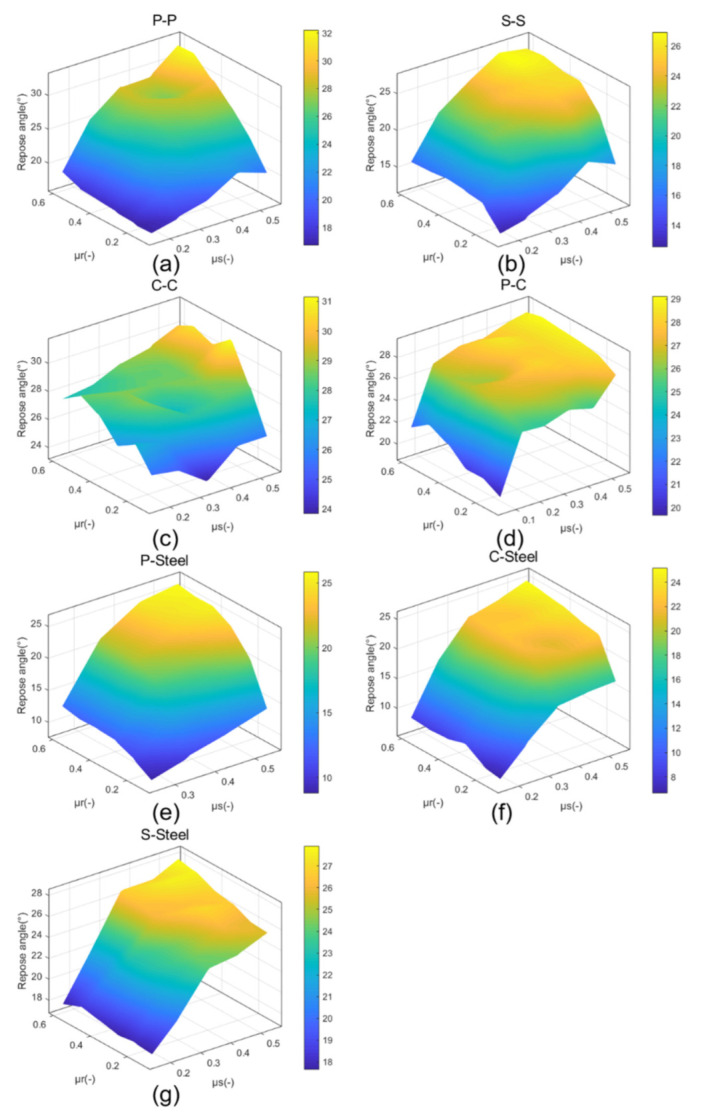
3D graphs of the relationship of repose angle, CORF and COSF of experiment groups: (**a**) P-P, (**b**) S-S, (**c**) C-C, (**d**) P-C, (**e**) P-Steel, (**f**) S-Steel, and (**g**) C-Steel.

Figure 6 shows the repose angle under different CORF ($\mu_{r}$) and COSF ($\mu_{s}$) of the simulation groups. It can be seen from the graphs that with the increase of CORF and COSF, the repose angle also increases. Figure 7 depicts 3D graphs of the relationship between the repose angle and CORF and COSF, showing that the slope of is initially steep and then gradually levels out. Furthermore, Figure 6 and Figure 7 indicate that the repose angle of each group from big to small are P-P > C-C > P-C > C-Steel > P-Steel > S-S > S-Steel.

Comparing the particle–particle group and particle–steel plate group, the repose angle of the particle–particle group is greater than that of the particle–steel plate group with the same CORF and COSF: a large roughness of the base surface will make the displacement shorter with the same path, thereby obtaining a greater repose angle. This causes the repose angle of the particle–particle group to be larger.

#### 3.1.3. Relationship between CORF and Repose Angle

Figure 6 and Figure 7 illustrate that as CORF increases, the repose angle increases. Comparing the graphs in Figure 6, it can be seen that there is a difference in the growth trend for different experimental groups. Comparing (a), (b), (e), and (f) with (c), (d), and (g) in Figure 6, the former group is seen to show a larger increase. By analyze the system, it was found that the latter groups contain coke, which has a lower density than pellet and sinter. It is known that a collapse of a particle pile depends on gravity and friction. The smaller the density of the particles, the smaller the friction it bears. Therefore, parameters other than CORF and COSF are also important for the repose angle.

Figure 6 and Figure 7 also show that when CORF of the group increases from 0.4 to 0.6, the repose angle increased only slightly or even occasionally decreased. This agrees with the findings of Coetzee [8]. It is believed that with the increase of CORF, the rolling of the particles become more and more difficult during the collapse of the particle pile. This means that the particles tend to slide more, and the rolling distance of the particles will continue to decrease. This phenomenon can simply be expressed as ${W=\mu }_{r}NX$, where *W* is the work done by the particle, *X* is the particle rolling distance, and *N* is the force perpendicular to the rolling surface. With the increase of $\mu_{r}$, *X* tends to decrease. The decrease of the *W* in this process is because the increase of $\mu_{r}$ has less influence on *W* than on *X*.

#### 3.1.4. Relationship between COSF and Repose Angle

Figure 6 and Figure 7 suggest that the repose angle of the particle pile changes a lot with the change of COSF. The repose angle difference between adjacent COSF is generally greater than 1° and even up to 10°, while for CORF, it is generally less than 1°, and the maximum is 6.9°. Another finding is that the relationship between CORF and the repose angle is affected by COSF. Figure 6a confirms that the repose angle will increase with the increase of CORF, especially for COSF > 0.15, but the change in repose angle is small when COSF ≤ 0.15. Particles with low COSF more easily slide and usually have high speed, resulting in a short contact time of the particles and little influence of CORF. These two points support the theory that COSF has a greater impact on the repose angle of the particle pile than CORF. This result agrees with findings reported in the literature [41,42]: during the formation of the particle pile, static friction is ubiquitous no matter if the particles move from a static state, or if static particles contact with dynamic particles.

Figure 6a indicates that when COSF ≥ 0.35, the difference in repose angle between different COSF is generally less than 2°, but when COSF < 0.35, it is generally greater than 3°. This substantiates that after COSF ≥ 0.35, the degree of increase in the repose angle becomes smaller as the COSF increases, since the particles with high COSF tend to roll rather than slide. This is phenomenon can also be seen from Figure 6b–g and Figure 7.

### 3.2. Comparison between Simulation and Physical Experiment

For physical experiments, the results obtained after post-processing are shown in Table 4, where the reported repose angle is the average value of the repeated experiments. Considering that the measurement error of the repose angle is generally about 1°, and its relative standard deviation is within acceptable limits (≤0.1), these demonstrates the reliability of the results.

The repose angle obtained from the groups of physical experiment are in an order of C-C > C-Steel > S-S > P-C > P-P > S-Steel > P-Steel. Among them, only P-Steel is lower than 20°, and the repose angle of P-P is 7° larger than that of P-Steel. This is because the steel surface is relatively smooth. It can be seen that CORF and COSF between particle–particle are greater than that of particle–steel plate. Next, we make a comparison of the repose angle of the pile in the experiments and simulations to obtain suitable simulation parameters.

**Figure 8:**
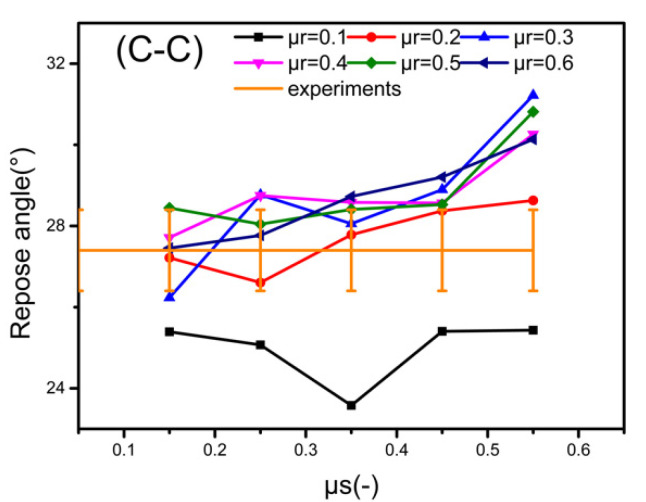
Choice of CORF and COSF in the intersecting part of the error range of the experimental line and the simulation lines.

**Figure 9:**
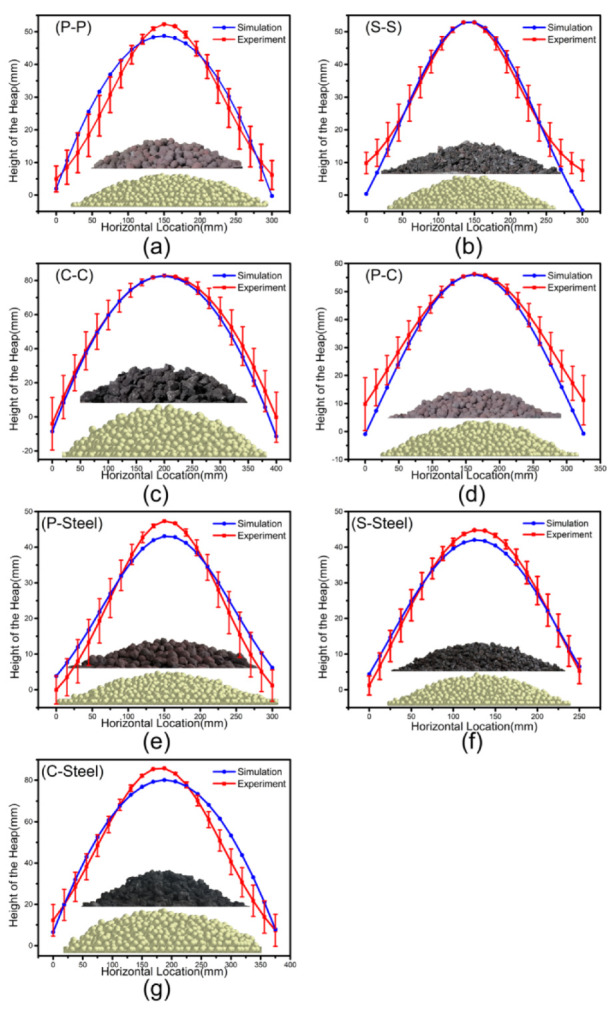
Comparison of bell curve of physical experiments and simulations: (**a**) P-P, (**b**) S-S, (**c**) C-C, (**d**) P-C, (**e**) P-Steel, (**f**) S-Steel, and (**g**) C-Steel.

To choose the appropriate CORF and COSF from the simulation results, as shown in Figure 8, we add a physical experiment line to Figure 6, and select the friction coefficient at the intersection point or within the range of the experiment line about 1°. The rules to determine the proper values (intersection point) of CORF and COSF are as follows: 1. In general, COSF should be larger than CORF. 2. CORF and COSF of particle–particle are greater than that of particle–steel plate. 3. The contours of the particle piles for simulations and physical experiments need to be similar. For CORF and COSF that meet the first two conditions, we use Matlab and the data processing method of Gaussian fitting to extract the fitting curve of particle pile surface to determine whether it meets the third point. Regarding the selection of the contour line fitting equation, although some investigators [43] describe the contour of the particle pile by a triangle, the contours observed in the experiments [29] are more irregular. The literature [37] proposes that it is more appropriate to describe the contour of this type of particle piles with Gaussian fitting, because it approximates over all data points and shows less errors. The CORF and COSF showing the best fit are reported in Table 5, and a comparison of their bell curves with the experiments is given in Figure 9.

Figure 9 compares the results of physical experiments and simulations with proper friction coefficients. The inserted figures show the simulated and experimental piles. In Figure 9a, the physical experiment curve is somewhat steeper than the simulated one, but the simulated curve falls within the error line, which means that the fit is good. Similarly, by studying Figure 9b–g, it is seen that the simulation and physical experiment curves basically overlap.

It is worth noting that Table 5 indicates that the values of CORF and COSF for coke and sinter in the particle–particle group and the particle–steel plate group show small differences, and the repose angle of the two groups in the physical experiments are also similar, as seen in Table 4. The particles move more easily on the steel plate than on the particles, so a smaller repose angle should be obtained when a particle pile is formed on the steel plate. Our results show that for non-spherical particles, the difference in the movement of the particles on the particles or steel plate is not very obvious, which ultimately leads to similar values of CORF and COSF between particle–steel plate and particle–particle.

In summary, it can be concluded that value of CORF and COSF can be determined by the outlined procedure, which is a basis for reliable DEM simulations of the behavior of the burden under charging and descent in the blast furnace.

## 4. Summary

In this paper, we applied DEM to simulate the formation of piles of pellet, sinter and coke particles, and studied the influence of the coefficients of rolling friction (CORF) and static friction (COSF) for particle–particle and particle–steel plate contact on the repose angle of the piles. The findings of the work generally agree with what has been reported by other investigators and the simulations were able to reproduce the experimental results. However, some differences between the present results and findings reported by other investigators were observed for the particle–particle and particle–steel plate friction coefficients of pellet, sinter and coke. The main conclusions of the present study are:

(1) The repose angle increases with CORF and COSF, but the growth rate gradually decays or eventually even becomes negative for CORF ≥ 0.4 or COSF ≥ 0.35.
(2) COSF has generally a greater effect than CORF on the repose angle.
(3) The rougher the base surface, the larger is the repose angle of the particle pile formed, as a rough base surface reduces the displacement of the particles through the same path, thereby forming a higher particle pile.
(4) The outlined procedure of fitting the contours of DEM-simulated piles with the experimental counterparts illustrate that appropriate CORF and COSF can be determined, which can be used to simulate the complex behavior of the burden in the ironmaking process.

## Figures and Tables

**Table 1:** Physical experiments particle parameters.

Materials	Pellet	Sinter	Coke
Mass (kg)	4	2	2
Size (diameter/ESD, mm)	8–13	6–8	13–15
Particle number (-)	2000	2500–3500	1500–2000

**Table 2:** Simulation parameters of pellet, sinter, coke and steel plate.

Materials	P	S	C	Steel
Diameter (mm)	10	8	13	
Density (kg/m^3^)	2284	3300	1050	7800
Young’s modulus (Pa)	2.5 × 10^7^	3.5 × 10^9^	5.37 × 10^8^	2 × 10^11^
Poisson’s ratio (-)	0.25	0.25	0.22	0.30
Coefficient of restitution (-)	0.60	0.18	0.20	

**Table 3:** Coefficient of restitution, COSF and CORF in particle–particle and particle–steel plate.

Groups	P-P	S-S	C-C	P-Steel	S-Steel	C-Steel	P-C
Coefficient of restitution (-)	0.42	0.35	0.39	0.62	0.40	0.42	0.40
COSF (-)	0.65	0.76	0.87	0.36	0.52	0.50	
CORF (-)	0.24	0.38	0.46	0.16	0.25	0.31	

**Table 4:** Measurement results of the repose angle of physical experiment and its relative standard deviation.

Groups	P-P	S-S	C-C	P-C	P-Steel	S-Steel	C-Steel
Repose angle (°)	24.73	24.96	27.40	24.91	17.68	23.72	27.22
Relative standard deviation (-)	0.086	0.095	0.049	0.044	0.075	0.063	0.086

**Table 5:** Comparison of repose angle between physical experiments and simulations.

Groups	Physical/°	Simulation/° (*μ_s_*, *μ_r_*)	Relative Difference/°
P-P	24.73	25.06 (0.55, 0.20)	0.33
S-S	24.96	25.53 (0.50, 0.30)	0.57
C-C	27.40	28.89 (0.45, 0.30)	1.49
P-C	24.91	25.60 (0.25, 0.10)	0.69
P-Steel	17.68	17.87 (0.45, 0.20)	0.19
S-Steel	23.72	23.71 (0.50, 0.20)	0.01
C-Steel	27.22	27.32 (0.42, 0.25)	0.10

## Data Availability

Data are contained within the article and can be requested from the corresponding author.
